# Telmisartan directly ameliorates the neuronal inflammatory response to IL-1β partly through the JNK/c-Jun and NADPH oxidase pathways

**DOI:** 10.1186/1742-2094-9-102

**Published:** 2012-05-29

**Authors:** Tao Pang, Juan Wang, Julius Benicky, Enrique Sánchez-Lemus, Juan M Saavedra

**Affiliations:** 1 Division of Intramural Research Programs, National Institute of Mental Health, National Institutes of Health, Department of Health and Human Services, Section on Pharmacology, NIMH, NIH, DHHS, 10 Center Drive, Bldg. 10, Room # 2D-57, Bethesda, MD, 20892, USA

**Keywords:** Angiotensin II AT_1_ receptor blockers, SK-N-SH neuroblasts, Cortical neurons, Neuronal inflammation, Neuroprotection, Oxidative stress, COX-2, PGE_2_ release, JNK activation, IL-1β neurotoxicity

## Abstract

****Background**:**

Blockade of angiotensin II type 1 (AT_1_) receptors ameliorates brain inflammation, and reduces excessive brain interleukin-1 beta (IL-1β) production and release from cortical microglia. The aim of this study was to determine whether, in addition, AT_1_ receptor blockade directly attenuates IL-1β-induced inflammatory responses in neuronal cultures.

****Methods**:**

SK-N-SH human neuroblasts and primary rat cortical neurons were pretreated with telmisartan followed by exposure to IL-1β. Gene expression was determined by reverse transcriptase (RT)-PCR, protein expression and kinase activation by western blotting, NADPH oxidase activity by the lucigenin method, prostaglandin E_2_ (PGE_2_) release by enzyme immunoassay, reactive oxygen species (ROS) generation by the dichlorodihydrofluorescein diacetate fluorescent probe assay, and peroxisome proliferator-activated receptor gamma (PPARγ) involvement was assessed with the antagonists GW9662 and T0070907, the agonist pioglitazone and the expression of PPARγ target genes *ABCG1* and *CD36*.

****Results**:**

We found that SK-N-SH neuroblasts expressed AT_1_ but not AT_2_ receptor mRNA. Telmisartan reduced IL-1β-induced cyclooxygenase-2 (COX-2) expression and PGE_2_ release more potently than did candesartan and losartan. Telmisartan reduced the IL-1β-induced increase in IL-1R1 receptor and NADPH oxidase-4 (NOX-4) mRNA expression, NADPH oxidase activity, and ROS generation, and reduced hydrogen peroxide-induced COX-2 gene expression. Telmisartan did not modify IL-1β-induced ERK1/2 and p38 mitogen-activated protein kinase (MAPK) phosphorylation or nuclear factor-κB activation but significantly decreased IL-1β-induced c-Jun N-terminal kinase (JNK) and c-Jun activation. The JNK inhibitor SP600125 decreased IL-1β-induced PGE_2_ release with a potency similar to that of telmisartan. The PPARγ agonist pioglitazone reduced IL-1β-induced inflammatory reaction, whereas telmisartan did not activate PPARγ, as shown by its failure to enhance the expression of the PPARγ target genes *ABCG1* and *CD36*, and the inability of the PPARγ antagonists GW9662 and T0070907 to modify the effect of telmisartan on COX-2 induction. The effect of telmisartan on IL-1β-stimulated COX-2 and IL-1R1 mRNA expression and ROS production was replicated in primary rat cortical neurons.

****Conclusions**:**

Telmisartan directly ameliorates IL-1β-induced neuronal inflammatory response by inhibition of oxidative stress and the JNK/c-Jun pathway. Our results support the hypothesis that AT_1_ receptor blockers are directly neuroprotective, and should be considered for the treatment of inflammatory conditions of the brain.

## **Background**

Interleukin-1 beta (IL-1β) is a neuromodulator primarily synthesized by microglia, with multiple physiological roles including regulation of sleep, memory, synaptic plasticity, and the innate immune response [1-4]. IL-1β is also a powerful inflammatory cytokine [4]. Excessive IL-1β production and release injures neurons, and is considered a major factor in the development and progression of neurodegenerative disorders, stroke, brain injury, and depression [5-10]. At present, there are no effective treatments to control excessive neuroinflammation [11]. The search for novel, safe, and effective central anti-inflammatory drugs, including those directly antagonizing the IL-1β-induced neuronal injury [12,13], is therefore of major interest.

The brain renin–angiotensin system (RAS) has emerged as a novel therapeutic target. Increased RAS activation, leading to excessive AT_1_ receptor stimulation, is a major factor in the development and progression of brain inflammation as a consequence of central or systemic infection [14,15], heart failure [16], and aging [17]. In turn, administration of AT_1_ receptor blockers (ARBs) decreases brain inflammation and is neuroprotective [18,19]. Therapeutic effects of ARBs have been shown in rodent models of systemic inflammation [15], hypertension, cerebral ischemia and stroke [20-27], intracerebral hemorrhage [28], multiple sclerosis [29], Parkinson’s disease (PD) [30,31], Alzheimer’s disease (AD) [32,33], and aging [17]. The neuroprotective effect of ARBs, as reported in rodent models, is partly direct and not entirely dependent on its effects on cardiovascular regulation [15,22,23,32,33]. This neuroprotective effect has also been shown *in vitro* using neuronal cultures [15,34-36].

To further clarify the mechanisms of the direct anti-inflammatory effects of ARBs in neuronal targets, we studied the effects of ARBs in a well-characterized human neuronal system widely used as an *in vitro* model of neuronal injury, the SK-N-SH neuroblastoma cell line [37,38]. In particular, we focused on telmisartan as an ARB prototype because of its reported pleiotropic anti-inflammatory effects as an AT_1_ receptor antagonist and a peroxisome proliferator-activated receptor gamma (PPARγ) agonist [23,32,39-41]. We investigated whether telmisartan ameliorates the inflammatory response to IL-1β in SK-N-SH neuroblasts and what are the mechanisms involved in these effects, and we compared the effects of telmisartan in SK-N-SH neuroblasts with those in rat primary cortical neurons.

## **Methods**

### **Materials and reagents**

Cell-culture media and supplements were obtained from Invitrogen (Carlsbad, CA, USA). Recombinant rat IL-1β was purchased from R&D Systems (Minneapolis, MN, USA). Telmisartan, losartan, CGP 42112, PD 123319, pioglitazone, diphenyleneiodonium chloride (DPI), SP600125, GW9662 and T0070907 were all purchased from Sigma-Aldrich (St. Louis, MO, USA). Candesartan was a kind gift from Astra-Zeneca (Mőlndal, Sweden). Angiotensin II was purchased from Bachem (Torrance, CA, USA). Primers for real-time PCR were synthesized by BioServe (Beltsville, MD, USA). SYBR Green PCR Master Mix for qPCR was purchased from Applied Biosystems (Foster City, CA, USA). The remaining reagents for RNA isolation and reverse transcription were from Invitrogen. Primary antibodies used for western blot analysis were: rabbit polyclonal anti-nuclear factor-kappa B (NF-κB)-p65 antibody (1:2000, Millipore, Billerica, MA, USA); mouse polyclonal anti-cyclooxygenase-2 (COX-2) (1:1000, Cayman Chemical, Ann Arbor, MI, USA); rabbit anti-phospho-p38 mitogen-activated protein kinase (MAPK) (1:1000), rabbit anti-phospho-extracellular signal-regulated kinases (ERK)1/2 (1:1000), rabbit anti-phospho-JNK (1:1000), rabbit anti-phospho-c-Jun (1:1000), rabbit anti-IκB-α (1:1000), rabbit anti-β-actin (1:1000), and rabbit anti-histone H4 (1:1000), all from Cell Signaling Technology (Danvers, MA, USA). Secondary horseradish peroxidase-conjugated antibodies for western blot analysis were: donkey anti-rabbit IgG (1:5000, Amersham BioSciences, Piscataway, NJ, USA) and goat anti-mouse IgG (1:10,000, Jackson ImmunoResearch, West Grove, PA, USA). Protease inhibitor cocktail and SuperSignal West Dura Substrate for chemiluminescent detection were purchased from Thermo Fisher Scientific (Pittsburg, PA, USA). All other chemicals were obtained from Sigma-Aldrich unless otherwise stated.

### **SK-N-SH neuroblast culture**

Human SK-N-SH neuroblasts were obtained from the American Type Culture Collection (HTB-11, Rockville, MD, USA) and grown in MEM with Earle’s salts and HEPES, supplemented with 10 % fetal bovine serum and 100 U/ml penicillin/streptomycin. Cells were cultured at 37°C in a humidified atmosphere of 5 % CO_2_/95 % air until they reached 80 % confluence, then confluent monolayers were passaged routinely by trypsinization. Cells between passages 3 and 10 were used in this study, and before each experiment, they were starved overnight in a serum-free medium.

### **Primary rat cortical neuron culture**

All animal care and experimental procedures in the present study were approved by the National Institute of Mental Health Animal Care and Use Committee (Bethesda, MD, USA). All efforts were made to minimize the number of animals used and their suffering (*National Institutes of Health Guide for the Care and Use of Laboratory Animals*, Publication number 80–23, received 1996). Primary cortical neuron cultures were obtained from fetal Sprague–Dawley rats (Charles River Laboratories, Wilmington, MA USA) at embryonic day 18 (E18) [42]. Fetal cerebral cortices were collected and placed in ice-cold Hank’s balanced salt solution. After removal of the meninges, the cortices were dispersed into the same buffer containing 0.25 % trypsin, and digested for 15 minutes at 37°C. Trypsin digestion was stopped by adding a two-fold volume of DMEM, supplemented with 10 % FBS and 0.1 mg/ml DNase I. After gentle trituration, digested tissues were separated by centrifugation at 200 × *g* for 5 minutes. The cell pellets were resuspended in complete Neurobasal culture medium supplemented with 2 % B27 and 0.5 mmol/l GlutaMax. After filtration through a 70 μm cell restrainer (BD Falcon, Vernon Hills, IL, USA), cells were plated at a density of 1 × 10^6^ cells/ml onto poly-D-lysine coated plates (Becton Dickinson and Co., Franklin Lakes, NJ, USA). Cultures were incubated in a humidified atmosphere of 5 % CO_2_/95 % air at 37°C. Only mature cultures (10–14 days *in vitro*) were used in this study. Immunocytochemical validation with anti-microtubule-associated protein 2 (MAP-2) antibody and 4',6-diamidino-2-phenylindole (DAPI) showed that more than 95 % of the cells in the culture system were neurons (data not shown).

### **Drug treatment**

The cells were pre-incubated for 2 hours with telmisartan, candesartan, losartan, CGP 42112, PD 123319, DPI, SP600125, pioglitazone, T0070907, GW9662, or vehicle before exposure to IL-1β. Most of the experiments were performed with the maximum stimulatory concentration of 10 ng/ml IL-1β, and the exposure times were 2 hours for ROS determination, 3 hours for RT-PCR analysis, and 24 hours for COX-2 protein and PGE_2_ determinations. The SK-N-SH neuroblasts were incubated with 100 μmol/l H_2_O_2_ for 3 hours to determine the protective effect of telmisartan. Activation of MAPKs, c-Jun, and NF-κB was determined by western blotting at various time intervals up to 2 hours. All concentrations used and time intervals are indicated in the figure legend for each particular experiment. All drugs were initially prepared as 1000-fold concentrated stock solutions, and were added directly into the cell-culture medium. Telmisartan, DPI, SP600125, pioglitazone, T0070907, and GW9662 were dissolved in dimethyl sulfoxide (DMSO). The final concentration of DMSO in experimental conditions was 0.1 %. Candesartan was initially dissolved in 0.1 mol/l Na_2_CO_3_, and further diluted to stock concentration with isotonic saline, at a final pH of 7.5 to 8.0. All other drugs were dissolved in isotonic saline. Control cells were treated with the corresponding vehicle in all experiments.

### **Real-time PCR**

Total RNA was isolated using TRIzol reagent followed by purification using an RNeasy Mini Kit (Qiagen, Valencia, CA, USA) in accordance with the manufacturer’s instructions. Synthesis of complementary DNA (cDNA) was performed with 0.6 μg of total RNA and Super-Script III first-Strand Synthesis Kit (Invitrogen, Carlsbad, CA, USA). Quantitative real-time PCR was performed on DNA Engine Opticon™ (MJ Research, Waltham, MA) with SYBR Green PCR Master Mix. PCR was performed in a 20 μl reaction mixture containing 10 μl SYBR Green PCR Master Mix, 2 μl cDNA and 0.3 μmol/l of each primer for a specific target (Table 1). The amplification conditions consisted of 1 denaturation/activation cycle at 95°C for 10 minutes, followed by 40 to 45 cycles at 95°C for 15 seconds and 60°C for 60 seconds. Serial dilutions of cDNA from the same source as samples were used to obtain a standard curve. The individual targets for each sample were quantified by determining the cycle threshold (Ct) and by comparison with the standard curve. The relative amount of the target mRNA was normalized to the level of GAPDH mRNA.

**Table 1:** List of PCR primers used in the study

**Gene**	**Accession number**	**Forward primer (5′→3′)**	**Reverse primer (5′→3′)**
hAT_1_	S77410	ACCGCCCCTCAGATAATGTAAG	TGAAGTGCTGCAGAGGAATGTT
hCOX-2	NM_000963	GATTGCCCGACTCCCTTGG	AACTGATGCGTGAAGTGCTG
hGAPDH	NM_002046	CCCATCACCATCTTCCAGGAG	GTTGTCATGGATGACCTTGGCC
hIκB-α	NM_020529	CGGACTGCCCTTCACCTC	ACATCAGCCCCACACTTCAA
hIL-1R1	NM_000877	AGAGGAAAACAAACCCACAAGG	CTGGCCGGTGACATTACAGAT
hNOX-1	NM_013955	ATCACAACCTCACCTTCCAC	ATAGGCTGGAGAGAATGGA
hNOX-2	NM_000397	CCCTTTGGCACTGCCAGTGAAGAT	CAATCCCTGCTCCCACTAACATCA
hNOX-4	NM_016931	GGATCACAGAAGGTTCCAAGCAG	GCAGCCACATGCACGCCTGAGAA
hNOX-5	NM_024505	ATCAAGCGGCCCCCTTTTTTTCAC	CTCATTGTCACACTCCTCGACAGC
rAT_1A_	NM_030985	AGCCTGCGTCTTGTTTTGAG	GCTGCCCTGGCTTCTGTC
rCOX-2	AF233596	CGGAGGAGAAGTGGGGTTTAGGAT	TGGGAGGCACTTGCGTTGATGG
rGAPDH	NM_017008	ATGACTCTACCCACGGCAAG	TGGAAGATGGTGATGGGTTT
rIL-1R1	NM_013123	TGAATGTGGCTGAAGAGCAC	CTTCCATCGTCTCATTCCGT

h, human; r, rat.

For AT_1_ receptor mRNA expression, the products of PCR amplification were separated on 3 % agarose gel and visualized with ethidium bromide to verify the size of amplicon.

### **Western blotting**

For the determination of NFκB-p65 nuclear translocation, nuclear protein extracts were prepared using Nuclear Extraction Kit (Pierce, Rockford, IL, USA) in accordance with the manufacturer’s instructions. For other proteins, the whole-cell lysates were prepared in Tris-Glycine SDS Sample Buffer (Invitrogen). The protein extracts were separated by electrophoresis on 10 % SDS-PAGE gels and transferred onto polyvinylidene fluoride (PVDF) membranes. The membranes were blocked for 1 hour and incubated overnight at 4°C with the primary antibodies, followed by washing and exposure to secondary antibodies for 1 hour at room temperature. The membranes were exposed to SuperSignal West Dura Substrate for chemiluminescent detection.

### **Measurement of reactive oxygen species**

The levels of intracellular ROS were determined by the change in the fluorescence resulting from the oxidation of the fluorescent probe H_2_DCFDA using OxiSelect™ ROS Assay Kit (Cell Biolabs, San Diego, CA, USA) in accordance with the manufacturer’s instructions. After preincubation with telmisartan or DPI, the cells were loaded with H_2_DCFDA for 30 minutes at 37°C and exposed to IL-1β for an additional 2 hours. The level of fluorescence, corresponding to intracellular ROS, was determined using a plate reader (VICTOR3; Perkin-Elmer, Torrance, CA, USA) with 485 nm excitation and 535 nm emission filters.

### **Prostaglandin E**_**2**_**measurement by enzyme immunoassay**

PGE_2_ release was determined in cells culture medium by enzyme immunoassay (EIA) (PGE_2_ EIA Kit; Cayman Chemical) in accordance with the manufacturer’s instructions.

### **NADPH oxidase activity assay**

The lucigenin method was used to determine NADPH oxidase activity in SK-N-SH cells. Cells were collected by scraping, and pelleted by centrifugation at 500 × *g* for 5 minutes. The pellets were resuspended and homogenized in ice-cold buffer containing 50 mmol/l Tris, pH 7.4, 1 mmol/l EDTA, 1 mmol/l DTT, 0.5 mmol/l phenylmethylsulfonyl fluoride (PMSF) and 1× protease inhibitor cocktail. The crude membrane fraction was pelleted by centrifugation at 16,000 × *g* for 90 minutes at 4°C, and the pellets were resuspended in 200 μl of assay buffer containing 8 mmol/l sodium phosphate, pH 7.4, 140 mmol/l NaCl, 10 mmol/l KCl, 2 mmol/l MgCl_2_, 50 mmol/l triethanolamine, 1 mmol/l DTT, and 1× protease inhibitor cocktail. The total protein concentration was determined by the Bradford assay and adjusted to 1 mg/ml. An aliquot (200 μl) of protein sample (100 μg of membrane proteins) were incubated in the presence of 5 μmol/l lucigenin and 100 μmol/l NADPH. The luminescence was monitored at 2-minute intervals using a plate reader (VICTOR3; Perkin-Elmer) to determine relative changes in NADPH oxidase activity.

### **Ang II measurement by enzyme immunoassay**

Ang II concentration in the cell-culture medium was measured using a commercial kit (Ang II EIA Kit; Cayman Chemical) following the manufacturer’s instructions. The limit of sensitivity of the assay was 1.5 pg/ml.

### **Statistical analysis**

Statistical significance was determined using GraphPad Prism 5 Software (GraphPad Software, San Diego, CA, USA). Multiple group comparisons were performed by one-way ANOVA followed by Newman-Keuls Post test. Differences were considered significant at *P* < 0.05. Values are expressed as the mean ± SEM.

## **Results**

### **Dose response and time course of interleukin-1β-induced neuronal inflammatory response**

**Figure 1:**
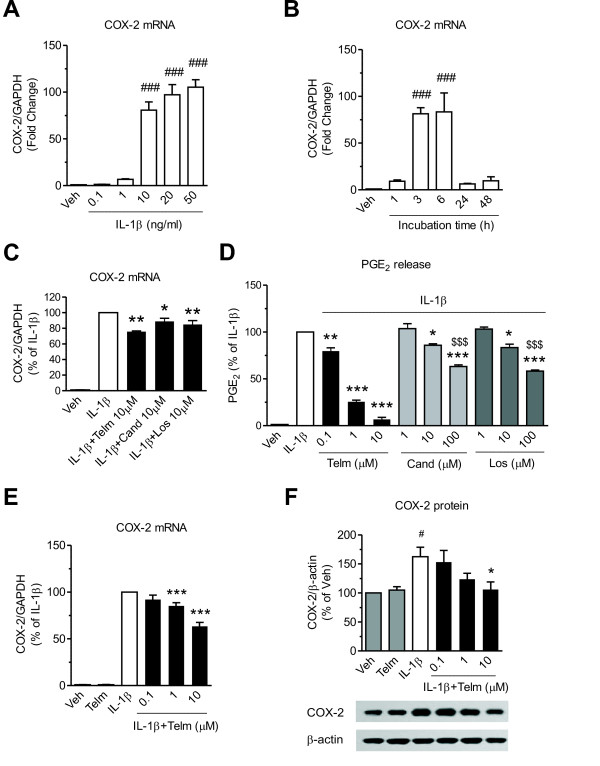
**Telmisartan inhibits interleukin-1 beta (IL-1β)-induced cyclooxygenase-2 (COX-2) mRNA and protein expression and prostaglandin E**_**2**_**(PGE**_**2**_**) release in SK-N-SH neuroblasts** (**A,B) IL-1β dose-dependently and time-dependently induces COX-2 mRNA expression in SK-N-SH neuroblasts.** The cells were incubated with **(A)** the indicated concentrations of IL-1β for 3 hours, or with **(B)** 10 ng/ml IL-1β at indicated time intervals to determine COX-2 mRNA expression. Results are expressed as fold change relative to vehicle-treated group (Veh). **(C)** Telmisartan, candesartan, and losartan reduced IL-1β induced COX-2 mRNA expression with similar potency. The cells were pretreated with 10 μmol/l telmisartan (Telm), candesartan (Cand), or losartan (Los) for 2 hours before exposure for 3 hours to 10 ng/ml IL-1β. Results are expressed as the percentage of IL-1β. **(D)** Telmisartan was the most effective of AT_1_ receptor blockers at reducing the IL-1β-induced PGE_2_ release. Cells were pretreated with indicated concentrations of Telm, Cand, or Los for 2 hours before exposure for 24 hours to 10 ng/ml IL-1β to determine cumulative PGE_2_ release. Results are expressed as the percentage of IL-1β. **(E,F)** Telmisartan dose-dependently reduced IL-1β-induced COX-2 mRNA and protein expression. The cells were pretreated with indicated concentrations of Telm for 2 hours, then incubated with 10 ng/ml IL-1β for **(E)** 3 hours to determine COX-2 mRNA expression, or **(F)** 24 hours to determine COX-2 protein expression. The picture is a representative western blot. All results are means ± SEM from at least three independent experiments. * *P* < 0.05, ** *P* < 0.01, *** *P* < 0.001 vs. IL-1β; # *P* < 0.05, ### *P* < 0.001 vs. Veh; $$$ *P* < 0.001 vs. IL-1β + 10 μmol Telm.

Incubation of SK-N-SH neuroblasts in the presence of IL-1β induced COX-2 mRNA expression in a dose-dependent and time-dependent manner (Figure 1A,B). Maximum stimulation of COX-2 mRNA was obtained with 10 ng/ml IL-1β, and it reached a peak after 3 hours of exposure (Figure 1A and 1B). Thus, this dose of IL-1β was selected for all subsequent experiments.

### **Angiotensin II receptor type 1 blockade reduces interleukin-1β-induced cyclooxygenase-2 expression and prostaglandin E**_**2**_**release**

Telmisartan, candesartan and losartan reduced IL-1β induction of COX-2 mRNA with equal potency (Figure 1C). All three ARBs dose-dependently reduced IL-1β-induced PGE_2_ release, but telmisartan was significantly more potent than candesartan or losartan (Figure 1D). Telmisartan dose-dependently decreased IL-1β-induced COX-2 mRNA expression (Figure 1E) and COX-2 protein expression (Figure 1F).

### **Angiotensin II receptor types in SK-N-SH neuroblasts and the effect of receptor blockade**

**Figure 2:**
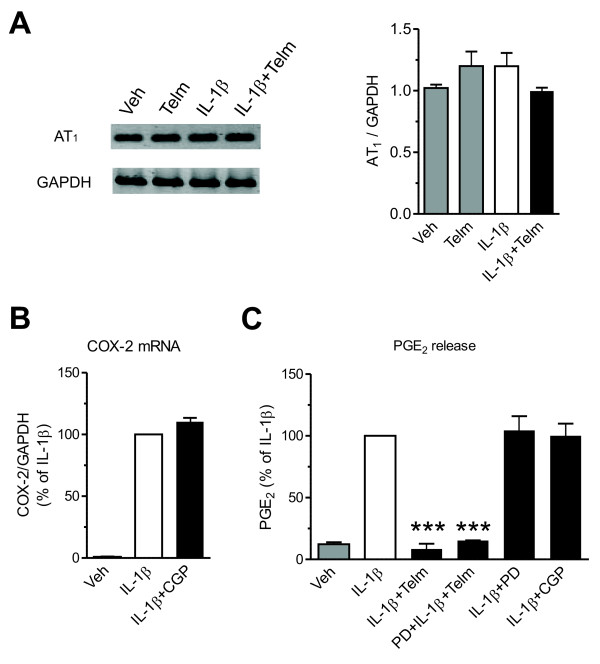
**Role of angiotensin II receptor type 1 (AT**_**1**_**) and type 2 (AT**_**2**_) **receptors in interleukin-1 beta (IL-1β)-induced neuronal inflammatory response in SK-N-SH cells. (A)** Expression of Angiotensin II AT_1_ receptor mRNA in SK-N-SH neuroblasts. The cells were pretreated with 10 μmol/l telmisartan (Telm) for 2 hours before exposure for 3 hours to 10 ng/ml IL-1β. (Right) AT_1_ receptor expression normalized to GAPDH mRNA. The picture shows visualized products of the RT-PCR reaction after separation on agarose gel. **(B)** Angiotensin II AT_2_ receptor agonist CGP 42112 (CGP) does not modify IL-1β-induced COX-2 mRNA expression. The cells were pretreated with 1 μmol/l CGP 42112 for 2 hours before exposur for 3 hourse to 10 ng/ml IL-1β. **(C)** Angiotensin II AT_2_ receptor antagonist PD 123319 or AT_2_ receptor agonist CGP 42112 do not affect IL-1β-induced PGE_2_ release or the inhibitory effect of telmisartan. The cells were pretreated for 2 hours with 10 μmol/l PD 123319 (PD), 10 μmol/l CGP, or 10 μmol/l Telm alone or in combination with PD, before exposure for 24 hours to 10 ng/ml IL-1β to determine cumulative PGE_2_ release. Results are expressed as a percentage of IL-1β. All results are means ± SEM from at least three independent experiments. *** *P* < 0.001 vs. IL-1β.

SK-N-SH neuroblasts expressed AT_1_ receptor mRNA, and the receptor expression was not affected by IL-1β or telmisartan, either alone or in a combination (Figure 2A).

AT_2_ receptor mRNA was not detectable in our preparation of SK-N-SH neuroblasts. Incubation in the presence of the AT_2_ receptor agonist CGP 42112 did not change IL-1β stimulation of COX-2 gene expression (Figure 2B) or PGE_2_ release (Figure 2C). Similarly, incubation in the presence of the AT_2_ receptor antagonist PD 123319 did not change IL-1β stimulation of PGE_2_ release, and did not alter the inhibitory effect of telmisartan (Figure 2C).

### **Telmisartan prevents interleukin-1β-induced NADPH oxidase activation, reactive oxygen species production and interleukin-1 receptor 1 gene expression**

**Figure 3:**
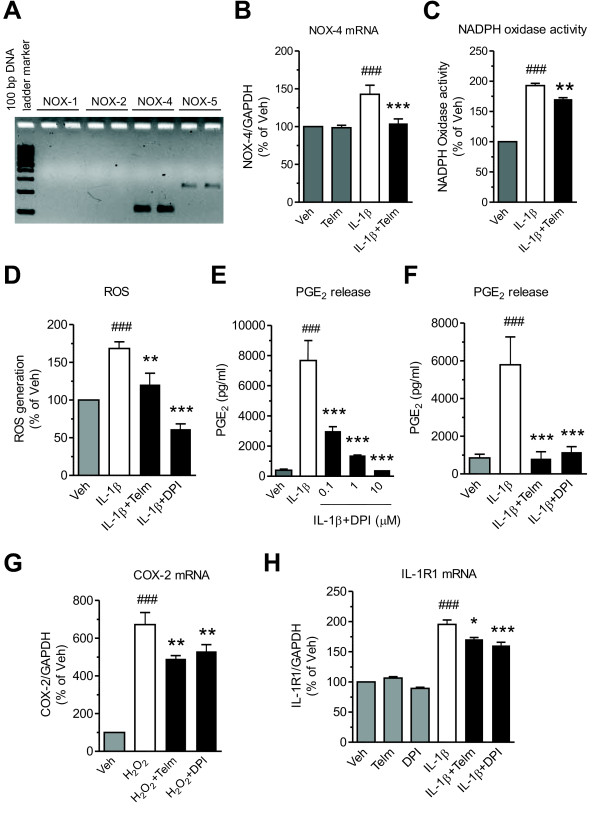
**Telmisartan reduces interleukin-1 beta (IL-1β)-induced NADPH oxidase activation, reactive oxygen species formation and IL-1 receptor 1 (IL-1R1) mRNA expression in SK-N-SH neuroblasts. (A)** Untreated SK-N-SH cells were analyzed for mRNA expression of different NADPH oxidase isoforms. NADPH oxidase-4 (NOX-4) is the dominant isoform of NADPH oxidase in SK-N-SH neuroblasts. The picture shows visualized products of a RT-PCR reaction after separation in an agarose gel. **(B,C)** Telmisartan reduced IL-1β-induced NOX-4 mRNA expression and NADPH oxidase activity. The cells pretreated with 10 μmol/l telmisartan (Telm) for 2 hours, were incubated with 10 ng/ml IL-1β for 3 hours to determine **(B)** NOX-4 mRNA expression and **(C)** NADPH oxidase activity**. (D)** Telmisartan reduced IL-1β-induced reactive oxygen species (ROS) generation to a lesser extent than does diphenyleneiodonium (DPI). The cells were pretreated with 10 μmol/l Telm or 5 μmol/l DPI for 2 hours before 1 hours exposure to 10 ng/ml IL-1β to determine ROS generation. **(E,F)** DPI dose-dependently inhibited IL-1β-induced PGE_2_ release with a potency similar to telmisartan. The cells pretreated with indicated concentrations of DPI or Telm for 2 hour were incubated with IL-1β for 24 hours to determine cumulative PGE_2_ release. **(G)** Both telmisartan and DPI reduce hydrogen peroxide-induced COX-2 mRNA expression. The cells were pretreated with 10 μmol/l Telm or 5 μmol/l DPI for 2 hours before exposure for 3 hours to 100 μmol/l hydrogen peroxide (H_2_O_2_) to determine COX-2 mRNA expression. **(H)** Both telmisartan and DPI reduce IL-1β-induced expression of IL-1β receptor IL-1R1 mRNA. The cells were pretreated with 10 μmol/l Telm or 5 μmol/l DPI for 2 hours before exposure for 3 hours to 10 ng/ml IL-1β to determine IL-1R1 mRNA expression. Results are presented as a percentage of Veh. All results are means ± SEM from at least three independent experiments. * *P* < 0.05, ** *P* < 0.01, *** *P* < 0.001 vs. IL-1β or H_2_O_2_; ### *P* < 0.001 vs. Veh.

High expression of the NADPH oxidase isoform NOX-4 and substantially lower expression of NOX-5 were found in SK-N-SH neuroblasts (Figure 3A). Expression of NOX-1 and NOX-2 was not detected (Figure 3A). Exposure to IL-1β significantly increased NOX-4 mRNA expression, and this effect was reduced by telmisartan (Figure 3B). IL-1β significantly increased NADPH oxidase activity, an effect also reduced by telmisartan (Figure 3C). IL-1β enhanced ROS production, and this effect was decreased by both telmisartan and DPI (Figure 3D). DPI dose-dependently inhibited IL-1β-induced PGE_2_ release (Figure 3E). The reduction in IL-1β-stimulated PGE_2_ release was similar for both telmisartan and DPI (Figure 3F).

Telmisartan reduced the enhanced COX-2 mRNA expression produced by H_2_O_2_ to an extent similar to that resulting from exposure to DPI (Figure 3G).

Exposure to IL-1β enhanced mRNA expression of its receptor, IL-1R1, and this change was reduced to a similar degree by telmisartan and DPI (Figure 3H).

### **Telmisartan decreases interleukin-1β-induced c-Jun N-terminal kinase and c-Jun activation**

**Figure 4:**
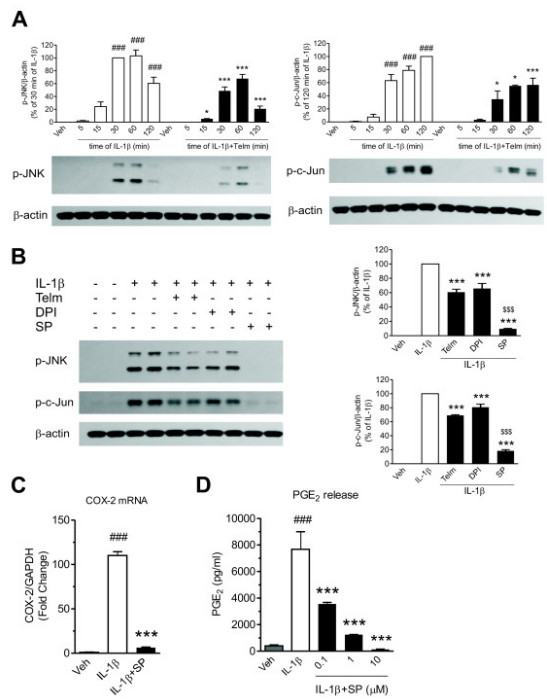
**Neuroprotective effect of telmisartan is partially mediated through inhibition of the c-Jun N-terminal kinase (JNK)/c-Jun pathway in SK-N-SH neuroblasts. (A)** Telmisartan attenuated the time-dependent activation of JNK and c-Jun in response to interleukin-1 beta (IL-1β). The cells were pretreated for 2 hours with 10 μmol/l telmisartan (Telm) before exposure to 10 ng/ml IL-1β for the indicated time intervals. Phosphorylation of JNK and c-Jun was determined by western blotting. Representative blots are shown under the corresponding bar graphs. ### *P* < 0.001 vs. Veh; * *P* < 0.05, *** *P* < 0.001 vs. corresponding IL-1β group. **(B)** Telmisartan inhibited IL-1β-stimulated JNK and c-Jun activation with a potency similar to that of diphenyleneiodonium (DPI) but to a lesser extent than the JNK inhibitor SP600125. The cells were pretreated for 2 hours with 10 μmol/l Telm, 5 μmol/l DPI, or 10 μmol/l SP600125 (SP) before exposure for 30 minutes to 10 ng/ml IL-1β. The phosphorylation of JNK and c-Jun was detected as above. Results are shown as a percentage of the IL-1β-treated group. **(C,D)** The JNK inhibitor SP600125 abrogated the IL-1β-induced COX-2 mRNA expression and PGE_2_ release. The cells were pretreated for **(C)** 2 hours with 10 μmol/l SP before exposure for 3 hours to 10 ng/ml IL-1β to determine COX-2 mRNA expression, or with **(D)** the indicated concentrations of SP600125 before exposure for 24 hours to 10 ng/ml IL-1β to determine cumulative PGE_2_ release. All results are presented as means ± SEM from three independent experiments. *** *P* < 0.001 vs. IL-1β; ### *P* < 0.001 vs. Veh; $$$ *P* < 0.001 vs. IL-1β + Telm.

IL-1β time-dependently activated JNK in SK-N-SH neuroblasts, reaching maximum stimulation after 30 to 60 minutes of exposure, and this effect was significantly reduced by telmisartan (Figure 4A). Exposure to IL-1β simultaneously and time-dependently enhanced c-Jun phosphorylation, a change significantly decreased by telmisartan (Figure 4A). The effect of telmisartan was of similar magnitude to that of DPI (Figure 4B). Incubation in the presence of the specific JNK inhibitor SP600125 abrogated the IL-1β-induced phosphorylation of JNK and c-Jun (Figure 4B), COX-2 mRNA expression (Figure 4C), and PGE_2_ release, in a dose-dependent manner (Figure 4D).

### **Telmisartan does not affect the interleukin-1β-stimulated activation of p38 mitogen-activated protein kinase, extracellular signal-regulated kinase 1/2, or nuclear factor-κB activation**

**Figure 5:**
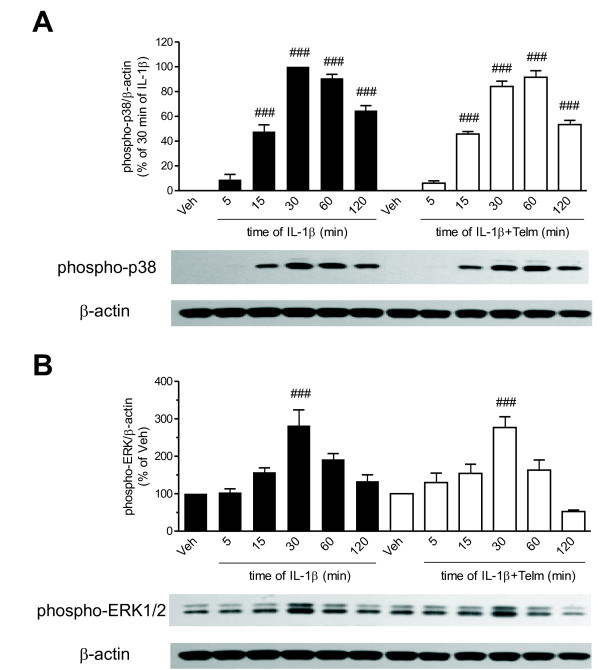
**Telmisartan did not alter the interleukin-1 beta (IL-1β)-stimulated activation of p38 mitogen-activated protein kinase (MAPK) or extracellular signal-regulated kinase (ERK)1/2 in SK-N-SH neuroblasts. (A,B)** Cells were pretreated for 2 hours with 10 μmol/l telmisartan (Telm) before exposure to 10 ng/ml IL-1β for the indicated time intervals. The phosphorylations of **(A)** p38 MAPK and **(B)** ERK1/2 were determined by western blotting and normalized to the levels of β-actin. All data are presented as means ± SEM from three independent experiments. Representative blots are shown under the corresponding bar graphs. ### *P* < 0.001 vs. Veh.

**Figure 6:**
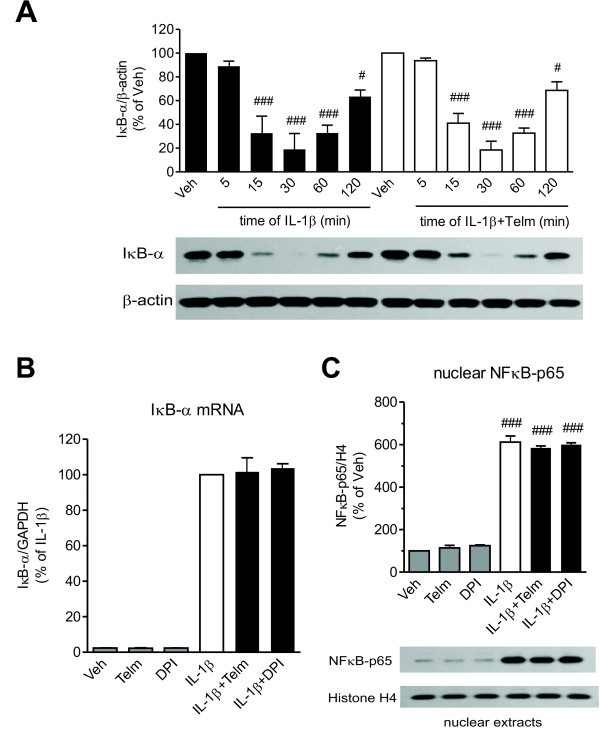
**The nuclear factor-kappa B (NF-κB) pathway is not involved in the neuroprotective effect of telmisartan in SK-N-SH neuroblasts. (A)** Telmisartan does not prevent time-dependent IκB-α protein degradation in cells in response to interleukin-1 beta (IL-1β). Cells were pretreated for 2 hours with 10 μmol/l telmisartan (Telm) before exposure to 10 ng/ml IL-1β for the indicated time intervals. IκB-α protein levels were determined in whole-cell extracts, and normalized to β-actin. **(B)** Neither telmisartan nor diphenyleneiodonium (DPI) modified IL-1β-induced expression of IκB-α mRNA. The cells were pretreated for 2 hours with 10 μmol/l Telm or 5 μmol/l DPI before exposure for 3 hours to 10 ng/ml IL-1β to determine IκB-α mRNA expression. **(C)** Neither telmisartan nor DPI affected IL-1β-induced nuclear translocation of the NF-κB p65 subunit. The cells were pretreated for 2 hours with 10 μmol/l Telm or 5 μmol/l DPI before exposure for 30 minutes to 10 ng/ml IL-1β. The NF-κB p65 subunit protein was determined in nuclear extracts and normalized to the level of the nuclear protein histone H4. Representative western blots are shown below the corresponding bar graphs. Results are presented as means ± SEM from three independent experiments. # *P* < 0.05, ### *P* < 0.001 vs. Veh.

Incubation in the presence of telmisartan did not modify IL-1β-induced p38 MAPK phosphorylation (Figure 5A) or the ERK1/2 phosphorylation (Figure 5B). Telmisartan did not change the time-dependent IL-1β-induced IκB-α degradation (Figure 6A), the IκB-α mRNA expression (Figure 6B), or the NF-κB-p65 protein nuclear translocation (Figure 6C). DPI was equally ineffective, and did not change IL-1β-induced IκB-α mRNA expression or the NFκB-p65 protein nuclear translocation (Figure 6B and 6C).

### **Peroxisome proliferator-activated receptor-γ is not involved in the neuroprotective effect of telmisartan**

**Figure 7:**
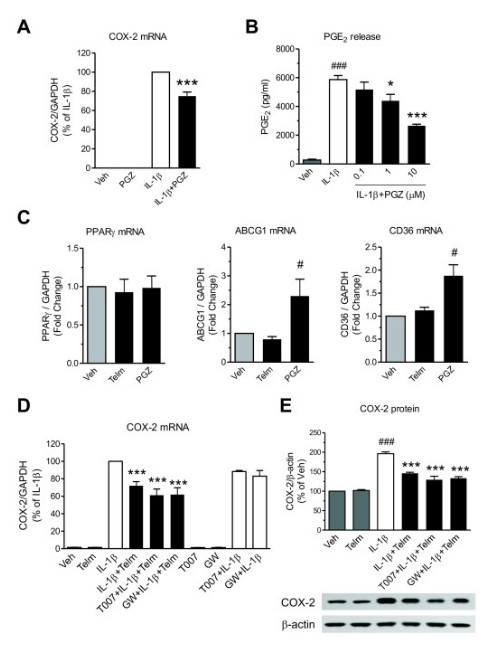
**Peroxisome proliferator-activated receptor gamma (PPARγ) activation is not involved in the neuroprotective effect of telmisartan in SK-N-SH neuroblasts. (A,B)** The PPARγ agonist pioglitazone inhibited interleukin-1 beta (IL-1β)-induced cyclooxygenase-2 (COX-2) gene expression and prostaglandin E_2_ (PGE_2_) release. The cells were pretreated for **(A)** 2 hours with 10 μmol/l pioglitazone (PGZ) before exposure for 3 hours to 10 ng/ml IL-1β to determine COX-2 mRNA expression or **(B)** with indicated concentrations of PGZ before exposure for 24 hours to 10 ng/ml IL-1β to determine cumulative PGE_2_ release. **(C)** Pioglitazone, but not telmisartan, induced gene expression of the PPARγ target genes *ABCG1* and *CD36*. The cells were incubated for 3 hours with 10 μmol/l PGZ or 10 μmol/l Telm to determine gene expression of PPARγ and its target genes *ABCG1* and *CD36*. Results are shown as fold change relative to the vehicle-treated group (Veh). **(D,E)** PPARγ antagonists did not change the inhibitory effect of telmisartan on IL-1β-induced COX-2 expression. The cells were pretreated for 1 hour with 1 μmol/l T0070907 (T007) or 20 μmol/l GW9662 (GW), followed by 10 μmol/l Telm for 2 hours before exposure for **(D)** 3 hours to 10 ng/ml IL-1β to determine COX-2 mRNA, or **(E)** 24 hours of IL-1β to determine COX-2 protein expression. The picture below is a representative western blot. All results are means ± SEM from at least three independent experiments. * *P* < 0.05, *** *P* < 0.001 vs. IL-1β; # *P* < 0.05, ### *P* < 0.001 vs. Veh.

Incubation of SK-N-SH neuroblasts with the PPARγ agonist pioglitazone significantly reduced IL-1β-induced COX-2 mRNA expression (Figure 7A), dose-dependently reduced PGE_2_ release (Figure 7B), and upregulated the mRNA expression of the PPARγ target genes *ABCG1* and *CD36*, without affecting PPARγ mRNA expression (Figure 7C). Conversely, telmisartan did not alter *ABCG1* or *CD36* mRNA expression (Figure 7C). Incubation of SK-N-SH neuroblasts in the presence of the PPARγ antagonists T0070907 or GW9662 alone did not significantly alter IL-1β-induced COX-2 mRNA expression (Figure 7D), and neither T0070907 nor GW9662 modified the inhibitory effect of telmisartan on IL-1β-induced COX-2 mRNA and protein expression (Figure 7D,E).

### **Effect of angiotensin II on the telmisartan neuroprotection in SK-N-SH neuroblasts**

**Figure 8:**
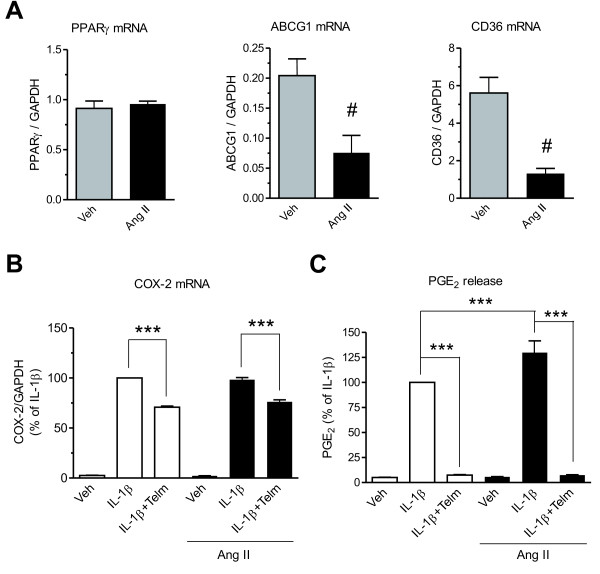
**Effect of Angiotensin II (Ang II) on SK-N-SH neuroblasts. (A)** Ang II did not affect peroxisome proliferator-activated receptor gamma (PPARγ) gene expression, but strongly inhibited the expression of the PPARγ target genes *ABCG1* and *CD36*. The cells were treated with 1 μmol/l Ang II for 24 hours to determine PPARγ, *ABCG1* and *CD36* mRNA expression. # *P* < 0.05 vs. Veh. **(B)** Ang II affected neither interleukin-1 beta (IL-1β)-induced cyclooxygenase-2 (COX-2) mRNA expression nor the inhibitory effects of telmisartan. Cells cultured for 24 hours in the presence of 1 μmol/l Ang II were pretreated for 2 hours with 10 μmol/l telmisartan (Telm) before exposure for 3 hours to 10 ng/ml IL-1β to determine COX-2 mRNA expression. Results are presented as a percentage of the IL-1β-treated group. **(C)** Ang II augmented IL-1β-induced PGE_2_ release but did not modify the inhibitory effect of telmisartan. The cells, cultured for 24 hours in the presence of 1 μmol/l Ang II, were pretreated for 2 hours with 10 μmol/l Telm before exposure for 24 hours to 10 ng/ml IL-1β to determine cumulative PGE_2_ release. Results are presented as a percentage of IL-1β. All results are means ± SEM from at least three independent experiments. *** *P* < 0.001.

Angiotensin II levels were undetectable in the cell-culture medium (results not shown). Exposure of SK-N-SH neuroblasts to 1 μmol/l Ang II for 24 hours did not alter PPARγ gene expression but strongly decreased gene expression of the PPARγ target genes *ABCG1* and *CD36* (Figure 8A). Pretreatment of neuroblasts with Ang II for 24 hours did not change basal COX-2 mRNA expression or basal PGE_2_ release. Ang II did not affect COX-2 mRNA expression induced by 10 ng/ml IL-1β, but did enhance IL-1β-induced PGE_2_ release (Figure 8B,C). Pretreatment with Ang II did not change the inhibitory effect of telmisartan on IL-1β-stimulated COX-2 gene expression and cumulative PGE_2_ release (Figure 8B,C).

### **Telmisartan reduces interleukin-1β upregulation of reactive oxygen species formation, interleukin-1 receptor type 1 and cyclooxygenase-2 mRNA expression in primary rat cortical neurons**

**Figure 9:**
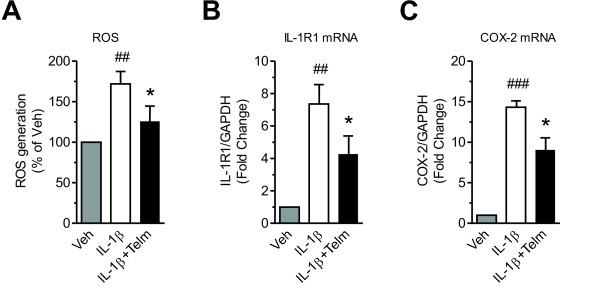
**Telmisartan inhibits interleukin-1 beta (IL-1β)-induced reactive oxygen species (ROS) generation, interleukin-1 receptor type 1 (IL-1R1) and cyclooxygenase-2 (COX-2) gene expression in primary rat cortical neurons.** Cells were pretreated for 2 hours with 10 μmol/l telmisartan (Telm) before exposure for 2 hours to 1 ng/ml IL-1β to determine **(A)** ROS generation, and **(B, C)** IL-1R1 and COX-2 mRNA expression. Results are means ± SEM from at least three independent experiments. * *P* < 0.05 vs. IL-1β; ## *P* < 0.01, ### *P* < 0.001 vs. Veh.

Exposure of primary rat cortical neurons to IL-1β induced both COX-2 and IL-1R1 mRNA expression and ROS generation, and these effects were significantly reduced by telmisartan (Figure 9A-C).

## **Discussion**

This study was designed to test the hypothesis that direct neuronal exposure to ARBs is neuroprotective. IL-1β was selected based on its well-characterized participation in neuronal injury associated with inflammatory and neurodegenerative diseases of the brain [6-10]. The principal finding of our study is that ARBs, in particular telmisartan, directly and significantly ameliorate the IL-1β-induced neuronal inflammatory response.

Ang II stimulates two receptor types, the AT_1_ and AT_2_ receptors [43]. Excessive AT_1_ receptor stimulation is associated with brain inflammation, whereas stimulation of AT_2_ receptors has been proposed to exert balancing neuroprotective effects, particularly when AT_1_ receptors are blocked by ARB administration [43-45]. SK-N-SH human neuroblasts expressed AT_1_ receptor mRNA, whereas AT_2_ receptor mRNA was undetectable in these cells. Furthermore, exposure of SK-N-SH neuroblasts to PD 123319 (an AT_2_ receptor antagonist) or CGP 42112 (an AT_2_ receptor agonist) did not change the effects of IL-1β, and PD 123319 did not modify the neuroprotective effect of telmisartan. These results indicate that the neuroprotective effect of telmisartan and other ARBs in SK-N-SH neuroblasts is dependent on AT_1_ receptor blockade without involvement of AT_2_ receptors.

The neurotoxic effects of IL-1β, confirmed in this study, have been well characterized. They depend on stimulation of the IL-1R1 receptor, and characteristically involve NADPH oxidase activation and ROS formation, COX-2 induction, and PGE_2_ production and release, leading to neuronal toxicity and apoptosis [5,37,38,46-49]. Our results support the hypothesis that IL-1β, when produced in excess by activated microglia, may directly generate further inflammatory cascades in neurons, contributing to their increased vulnerability to injury.

Telmisartan, at concentrations similar to those found in clinical studies [50], significantly reduced the neuronal inflammatory response induced by IL-1β. Most of the downstream pathways activated by IL-1β in the present study, including IL-1R1 receptor upregulation, are associated with NADPH oxidase activation [51,52]. This indicates that inhibition of NADPH oxidase activity by telmisartan is a major neuroprotective mechanism. Telmisartan decreased not only IL-1β-induced ROS formation but also H_2_O_2_-induced COX-2 expression, suggesting that reduction of the intracellular ROS and ROS-related downstream pathway [10] may be important for the neuroprotective effects of telmisartan. The wide-ranging anti-oxidant effects described here were similar to those reported previously in non-neuronal cell lines [53] and were of a potency similar to that of the NADPH oxidase and NOS inhibitor DPI [54,55]. These results are in agreement with observations showing that ARBs decrease NADPH oxidase activation associated with oxidative stress and neuronal apoptosis [36,56,57]. The neuroprotective effects of telmisartan were replicated in rat primary cortical neurons, indicating that they were not limited to responses only in the neuroblast preparations.

The discovery that telmisartan significantly prevents the IL-1β-induced upregulation of its receptor IL-1R1 in both SK-N-SH neuroblasts and rat primary cortical neurons is of major interest. Most of the IL-1β effects are mediated by IL-1R1 receptor stimulation. Administration of IL-1R1 receptor inhibitors seems to lead to amelioration of brain inflammation, and protection from stroke and traumatic brain injury, thus the development of novel IL-1R1 receptor inhibitors is the subject of active research [12,13]. For these reasons, our finding that telmisartan significantly prevents IL-1β induction of its receptor indicates an additional anti-inflammatory mechanism that might be of clinical value.

In agreement with previous observations [58], we found that IL-1β significantly stimulates a number of kinases, including p38 MAPK, ERK1/2, and JNK/c-Jun, and produces a notable activation of NF-κB in human SK-N-SH neuroblasts. Incubation in the presence of telmisartan significantly reduced IL-1β-induced JNK/c-Jun activation, but had no effect on activation of p38 MAPK, ERK1/2, and NF-κB. Stimulation of inflammatory cascades is to a considerable extent the result of activation of the transcription factor NF-κB [10]. Our observations are therefore no unexpected and concur with those of previous studies showing that anti-inflammatory mechanisms are cell-specific, depending on the inflammatory component and on the anti-inflammatory agent studied. In monocytes, macrophages, and microglia, NF-κB activation seems to be a major factor leading to inflammation and COX-2/PGE_2_ production [10,41,49]. However, in brain endothelial cell lines, several important components of the IL-1β-induced inflammatory response are independent of MAPK activity [9]. Moreover, glucocorticoids reduce IL-1β-induced inflammation in cells of neural origin by mechanisms independently of NF-κB [59]. These results and our present findings indicate that factors independent of NF-κB play a major role in the anti-inflammatory effect of ARBs in neurons.

All ARBs inhibit the Ang II-induced effects associated with stimulation of physiological AT_1_ receptors, but some ARBs, particularly telmisartan, are also partial PPARγ agonists [39,40]. Surprisingly in SK-N-SH neuroblasts, telmisartan failed to activate PPARγ. Furthermore, addition of PPARγ antagonists did not modify the neuroprotective effects of telmisartan, indicating that in these cells, AT_1_ receptor inhibition rather than PPARγ activation may be the primary mechanism for the direct anti-inflammatory effects of ARBs. These observations apparently contrast with the initial demonstration of PPARγ activation by telmisartan in cell culture [39,40], the PPARγ-associated anti-inflammatory effects of telmisartan in cultured human monocytes and THP-1 cells [41], and the PPARγ-activating neuroprotective effects of telmisartan shown *in vivo*[23,31,32].

It has been reported that although conventional PPARγ agonists can suppress expression of pro-inflammatory factors in primary microglia, they do not suppress expression of pro-inflammatory molecules in a microglial cell line expressing little or no PPARγ [60,61], and are not neuroprotective when applied to neurons [62]. In the SK-N-SH neuroblast preparations used in the present study, the PPARγ gene was expressed at relatively low levels compared with AT_1_ receptors (data not shown). However, in spite of the low PPARγ gene expression, a conventional PPARγ full agonist, pioglitazone [63], significantly activated PPARγ in SK-N-SH neuroblasts. Conversely, under identical experimental conditions, telmisartan was ineffective, indicating that PPARγ activation is neuroprotective but is not mediating the effects of telmisartan in SK-N-SH neuroblasts.

Indeed, the PPARγ agonist properties of individual ARBs seem to depend on the cell type studied and on the conditions of the experiments. Reports from cell-culture studies indicated that the PPARγ agonist effects of candesartan and losartan are not high [39,40]; however, losartan has been found to increase PPARγ activation in certain cell types [64,65], and long-term candesartan treatment upregulates PPARγ gene expression *in vivo* in adipose tissue [66]. Further studies are necessary to clarify the relative contribution of AT_1_ receptor blockade and the PPARγ agonist activity of ARBs in specific cell populations. Whether the PPARγ agonist effect of ARBs may be dependent on the degree of PPARγ gene expression remains an open question.

It is known that Ang II strongly inhibits PPARγ activation, an effect dependent on AT_1_ receptor stimulation, and the absence of Ang II may substantially stimulate PPARγ activity [45,67,68]. In accordance with this, addition of a high Ang II concentration decreased expression of PPARγ target genes in our study. However, in our studies, Ang II levels in the cell-culture media were below the 1.5 pg/ml (corresponding to 1.5 pmol/l) limit of detection. A concentration of 1 μmol/l of Ang II was required to produce a small increase in the IL-1β-induced PGE_2_ release, whereas it did not change COX-2 induction, had no effect on NADPH oxidase expression or activity (data not shown), and did not influence the protective effects of telmisartan. For these reasons, it is very likely that in SK-N-SH neuroblasts, the neuroprotective effects of telmisartan are independent of Ang II-mediated stimulation of AT_1_ receptors. Ligand-independent AT_1_ receptor activation has been reported previously in cardiomyocytes as a consequence of mechanical stress [69].

Based on the present results, we propose that, in SK-N-SH neuroblasts, the AT_1_ receptor may be constitutively active, and the neuroprotective effects of telmisartan and other ARBs may be the result of a decrease of such constitutive AT_1_ receptor activity. Recently, the constitutive activity of AT_1_ receptor has been reported under basal conditions *in vivo* even in the absence of Ang II [70]. However, there are no reports of ligand-independent activation or constitutive AT_1_ receptor activity in neurons, and the hypothesis of constitutively active neuronal AT_1_ receptors requires further confirmation.

Although it must be considered that neuronal cultures may not be representative of *in vivo* conditions, the SK-N-SH neuroblasts cultures are a good *in vitro* model to study the mechanisms of action responsible for direct ARB neuroprotection.

**Figure 10:**
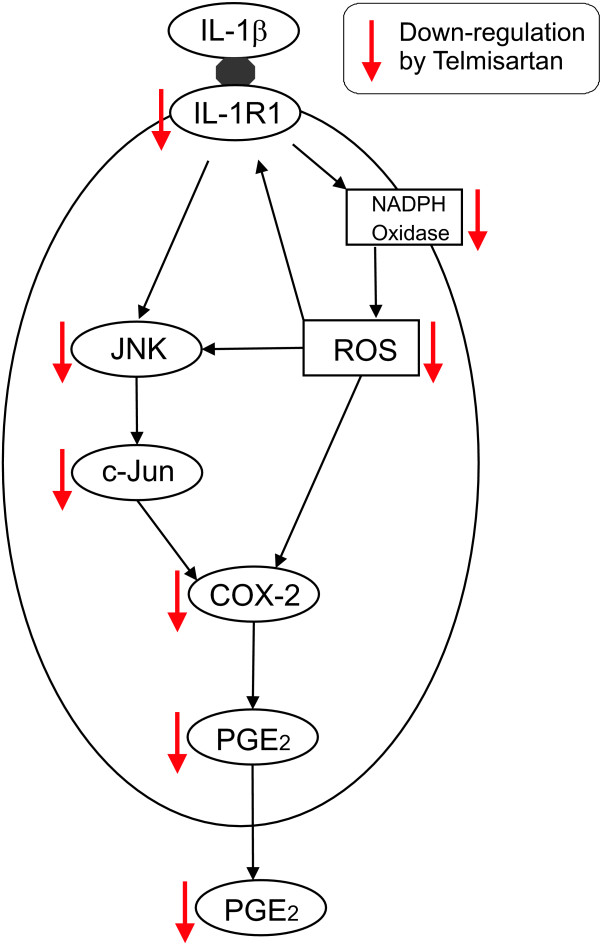
**Proposed pathways involved in the neuroprotective effects of telmisartan.** The pro-inflammatory effects of interleukin-1 beta (IL-1β) in neurons are the consequence of binding to its IL-1R1 receptor, which in turn increases NADPH oxidase activation, reactive oxygen species (ROS) formation, c-Jun N-terminal kinase (JNK) and c-Jun activation, and IL-1 receptor 1 (IL-1R1) gene expression. This results in enhanced expression of COX-2 and prostaglandin E_2_ (PGE_2_). Telmisartan abrogates these pro-inflammatory effects of IL-1β by mechanisms involving inhibition of NADPH oxidase activation and the JNK/c-Jun pathway.

The present observations and those of the literature suggest that ARBs may exert neuroprotective effects by several associated mechanisms: decreasing inflammation-induced circulating IL-1β levels affecting the brain and activating microglia in brain parenchyma, by direct anti-inflammatory effects in microglia as shown in isolated microglia in culture [15], and by direct effects in neurons, ameliorating the neuronal inflammatory responses produced by excess IL-1β, as reported here and illustrated in Figure 10.

Our results have important clinical implications. IL-1β is a strong stimulant of oxidative stress, COX-2 production, and PGE_2_ release, and it has been clearly associated with both acute and chronic inflammatory conditions of the brain. Neuronal induction of COX-2, leading to increased release of its product PGE_2_, is strongly stimulated by IL-1β, and has been linked to neuroinflammatory aspects of neurodegenerative diseases such as AD and HIV-associated dementia [38,71-73]. Furthermore, it was reported that maximal COX-2 expression predates maximal activation of astrocytes and microglia in the early stages of AD [74]. For this reason, the direct neuroprotective effects of ARBs reported here may be of major clinical significance.

Our present observations may explain the recent findings that ARB administration for the treatment of hypertension significantly protects cognition, and ameliorates the incidence and progression of AD, and that the neuroprotective effects of ARBs seem to be superior to those of similarly potent anti-hypertensive medications without direct effect on AT_1_ receptors [75,76]. These clinical observations are supported by pre-clinical studies, showing that ARBs reduce NADPH oxidase activation and neuronal apoptosis and protect cognition in animal models of AD and PD [36].

## **Conclusions**

Our observations highlight the pleiotropic neuroprotective effects of ARBs. As reported previously, these compounds reduce the inflammation-induced production of circulating inflammatory cytokines affecting the brain and inflammation-induced microglial activation, significantly diminishing inflammatory cascades. As we show here, ARBs directly decrease the pro-inflammatory effects of IL-1β in neurons, including reduction of IL-1β receptor upregulation, NADPH oxidase activation, ROS production, JNK and c-Jun activation, and pro-inflammatory COX-2/PGE_2_. We propose that ARBs may not only reduce production of excessive pro-inflammatory factors, but also decrease neuronal vulnerability to injury. These properties are of significant clinical value, and help to explain the increasing evidence that treatment with ARBs ameliorates the incidence and progression of acute and chronic neurodegenerative conditions such as AD and stroke, in which neuroinflammation plays an important role.

## Abbreviations

AD, Alzheimer’s disease; Ang II, Angiotensin II; ARB, Angiotensin II receptor blocker; AT1, Angiotensin II receptor, type 1; COX-2, Cyclooxygenase-2; DMEM, Dulbecco’s modified Eagle’s medium; DMSO, Dimethyl sulfoxide; DPI, Diphenyleneiodonium; EIA, Enzyme immunoassay; ERK1/2, Extracellular signal-regulated kinases 1/2; FBS, Fetal bovine serum; GAPDH, Glyceraldehyde 3-phosphate dehydrogenase; H2DCFDA, Dichlorodihydrofluorescein diacetate; IκB-α, Inhibitor of kappa B alpha; IL-1β, Interleukin-1 beta; IL-1R1, Interleukin 1 receptor 1; IL-6, Interleukin-6; JNK, c-Jun N-terminal kinase; MAPK, Mitogen-activated protein kinase; MEM, Minimum essential medium; NADPH, Nicotinamide adenine dinucleotide phosphate; NF-κB, Nuclear factor-kappa B; NOX, NADPH oxidase; PD, Parkinson’s disease; PGE2, Prostaglandin E2; PPARγ, Peroxisome proliferator-activated receptor gamma; RAS, Renin–angiotensin system; ROS, Reactive oxygen species; RT, Reverse transcriptase.

## **Competing interests**

The authors declare that they have no competing interests.

## **Authors’ contributions**

TP, JW, JB, and ES performed the experiments. TP, JMS conceived of and designed the experimental plan, and wrote the manuscript. All authors have read and approved the final version of the manuscript.
